# Sugarcane productivity and sugar yield improvement: Selecting variety, nitrogen fertilizer rate, and bioregulator as a first-line treatment

**DOI:** 10.1016/j.heliyon.2023.e15520

**Published:** 2023-04-14

**Authors:** Belete Desalegn, Erana Kebede, Hirpa Legesse, Tarekegn Fite

**Affiliations:** a College of Agriculture and Natural Resources, Wallaga University, P.O. Box: 395, Nekemte, Ethiopia; b School of Plant Sciences, College of Agriculture and Environmental Sciences, Haramaya University, P.O. Box: 138, Dire Dawa, Ethiopia; c Ethiopian Institute of Agricultural Research, Ambo Agricultural Research Centre, Ambo, Ethiopia

**Keywords:** Agrostemin, Cane yield, Crops®, Juice quality, Sugar yield

## Abstract

The improvement of sugarcane productivity depends on the crop varieties, growth environments, and management practices. In particular, the selection of the most productive variety and the use of an optimal fertilizer rate and plant bioregulator are critical for increasing sugarcane productivity and sugar yield. This study aimed to determine the high-performing sugarcane variety, optimal nitrogen rate, and potential bioregulator for improved sugarcane production, juice quality, and sugar yield. Two sugarcane varieties (D42/58 and NCo-334), three nitrogen (N) fertilizer rates (0, 100, and 150 kg/ha), and two bioregulators (Agrostemin and Crops®) were used for the study. The study revealed that sugarcane variety had a significant effect on all growth, yield, and quality parameters. Plant height was significantly influenced by variety and bioregulators, while stalk population was significantly influenced by two- and three-way interactions of varieties, bioregulators, and N fertilizer rates. Cane weight was significantly affected by variety, N fertilizer rate, and bioregulators, whereas cane yield was significantly affected by variety, N fertilizer, and their interaction effects. Sugar yield was similarly influenced by variety, bioregulator, and their interaction. The three main factors, as well as their two- and three-way interactions, had a considerable influence on cane quality parameters. Sugarcane variety D42/58 significantly outperformed variety NCo-334 in terms of sprouting, number of tillers, plant height, number of millable canes, and sucrose percentage by 7.49%, 9.50%, 12.80%, 10.50%, and 9.10%, respectively. The use of the D42/58 variety with N fertilizer (at 100 kg/ha) and/or the Agrostemin bioregulator also led to higher performance in cane population (107126), cane yield (153.34 tons/ha), Pol % (15.81%), and sugar yield (10.25 tons/ha). Most sugarcane growth, yield, juice quality, and sugar yield parameters were positively correlated; hence, high-performing varieties, appropriate N rates, and plant bioregulators could boost sugarcane productivity and sugar yield. Overall, the selection and combination of sugarcane variety D42/58 with 100 kg N/ha and/or Agrostemin bioregulator could maximize sugarcane production, juice quality, and sugar yield. To confirm the current findings, however, more research needs to be conducted across different agroecologies and seasons.

## 1 Introduction

Sugarcane (*Saccharum* spp. hybrid) is an important industrial crop used for sugar production, bioenergy, and other derivatives [[1], [2], [3], [4], [5]]. It is a monocotyledonous crop plant that is cultivated in the tropical and subtropical regions of the world, primarily for its ability to store a high concentration of sucrose or sugar in the internodes of the stem [6,7]. The sugarcane type grown today in different countries for the production of white sugar is *Saccharum hybrid*. This sugarcane type is getting worldwide attention due to its thick stalks, high sucrose content with a relatively soft rind, broad leaves, moderate tillering, reasonably erect stalks, and high cane yields [8,9]. Consequently, it is an economically important crop in tropical and subtropical regions, which is grown in 121 countries on 26.9 million hectares of land with a yield of 70.9 tonnes/hectare [2,10]. It is also the main sugar-producing crop that contributes nearly 70% of the total sugar pool at the global level followed by sugar beet (*Beta vulgaris* L.) [11].

In Ethiopia, the sugar industry utilizes only sugarcane and has great contributions to the socio-economy of the country [12,13]. Although the crop is not native to Ethiopia, it was grown in some parts of the country even before the commencement of large-scale commercial plantations and the establishment of a modern sugar factory at Wonji, mainly for local consumption [14]. The demand for sugar has increased significantly in developing countries like Ethiopia in the past few years [15,16]. For instance, Ethiopia's yearly per-person consumption of sugar has increased from 3.6 to 10 kg, but the country only produces 7 kg of it, and as a result, the remaining sugar is imported from other countries to meet the demand [17]. Consequently, developing countries like Ethiopia need to increase their sugar production to meet the domestic requirements of their increasing population and the economic growth of the nation [6,13]. However, sugarcane yield decline is now the main focus of attention in Ethiopian sugarcane production. Lack of proper management practices specific to fertilizers, improved varieties, and growth promoter treatments is among the major constraints of sugarcane production [[18], [19], [20], [21]].

Considering the sugarcane yield decline combined with changing and unpredictable climatic conditions, research focusing on the use of combined crop management practices such as high-performing varieties, optimal fertilizer rates, and plant bioregulators could better tackle multistress conditions [22]. The improvement of sugarcane production and sugar yield in particular depends on the crop varieties (genotypes), growth environments, and management practices, which in the first instance influence sugarcane yield and sucrose content [23,24]. Consequently, sugarcane cultivation utilizes various agro-inputs integrated into the production system, as well as new technologies emerging due to the research and advances in technology [25,26]. Earlier studies have reported that varieties of sugarcane differ in their responses to environmental conditions and various agro-input applications [27,28], especially in their uptake and utilization of various nutrients [26]. As a result, using sugarcane varieties with high adaptation, nutrient use efficiency, yield, and sucrose content has become an effective way to boost productivity, increase sugar yield, and reduce chemical fertilizer use [25]. Studies have also confirmed that sugarcane production often relies on the use of nitrogen (N) fertilizers, which improve agronomic parameters, juice quality, and sugar yield [1,25,29]. However, long-term excessive N fertilization led to a cost increment, high N losses, a reduction in N use efficiency, and negative environmental impacts [24,25]. Therefore, the application of a proper N fertilizer rate is beneficial for sugarcane's growth, development, and crop yield [25,30].

Efforts to improve sugarcane production through application of plant bioregulators are also getting attention due to their broad physiological role and yield increments under different conditions [22,31]. Plant bioregulators are hormone-like natural or synthetic compounds that are able to increase the yields and nutritional qualities, alter growth patterns, provide resistance to various stresses and improve soil productivity when applied at very low concentration to the plants and soils [32,33]. Compared to other agrochemical inputs, bioregulators are required in minor quantities, and the cost-benefit ratio is much higher [34]. As a result, bioregulators are now being used effectively in several countries to improve sugarcane yields and sugar content, thereby addressing the sugarcane cultivation crisis [35]. However, the use of bioregulators as agricultural inputs in Ethiopia is limited. In particular, information on the response of growth, yield, juice quality, and sugar yield of different sugarcane varieties, soil characteristics, and overall practical predictions for the co-application of N fertilizer and bioregulators under field conditions remain lacking in Ethiopia. Therefore, it is crucial to investigate how to optimize N fertilization co-applied with bioregulators to sustainably improve soil productivity, sugarcane productivity, and sugar production. This study aimed to evaluate the effects of sugarcane varieties, N fertilizer rates, and plant bioregulators on sugarcane productivity, cane juice quality, and sugar yield at Fincha'a Sugar Estate, Oromiya, Ethiopia.

## 2 Materials and methods

### 2.1 Description of the study site

The experiment was conducted during the cropping season of 2018/2019 at Fincha'a Sugar Estate, a recently established and modern sugar estate in Ethiopia, located in Abbay Comman district, Horro-Guduru Wallaga Zone, Oromiya Regional State, Ethiopia. The area is situated from 9° 30′ to 10^0^ 00′ N and 37° 15′ to 37° 30′ E. The elevation of Fincha'a Valley varies from 892 to 2520 m above sea level (m a.s.l.), while the altitudinal range of the study site is between 1350 and 1650 m a.s.l. (Kitila et al., 2015). The area lies within the drainage basin of the Nile Basin. The area's average annual rainfall is about 1315.38 mm and is categorized by a unimodal rainfall pattern with alternate wet and dry seasons, with significant rain falling between May and September. The average maximum and minimum daily temperatures are 30.6 and 14.5 °C, respectively [7]. The highest monthly average maximum temperature occurred in March (34.86 °C), and the lowest monthly average minimum temperature occurred in November (12.13 °C). The monthly average highest relative humidity (RH) is about 83.8%, and the average lowest RH is about 39%. The soils in the Fincha'a valley are made of alluvial and colluvial materials from the surrounding escarpments. The area's dominant soil types are Luvisols and Vertisols, which account for more than 95% of cultivated and irrigated lands [36].

### 2.2 Description of the planting materials

The high-yielding commercial sugarcane varieties at Fincha'a Sugar Estate, namely NCo-334 and D42/58, were used as planting materials. These varieties were selected based on their area of coverage and yield potential. The NCo-334 variety is a commercial sugarcane variety imported from India, and it is well grown on Vertisols at Fincha'a Sugar Estate and needs less water. The variety has a higher tillering capacity, erect leaves with straight stalks, many millable canes per hectare, and higher cane height than D42/58, but is less resistant to disease. The D42/58 variety is also a commercial sugarcane variety, having a higher sugar yield (ton per hectare) than the NCo-334 variety [37].

### 2.3 Description of bioregulators

Agrostemin® is a natural bioregulator obtained from the seeds of Corn Cockle (*Agrostemma Githago* L.), developed in Yugoslavia by Dr Danica Gajic. It is used worldwide as a bioregulator for several agricultural crops and has induced many favorable effects on plant growth, development, vegetative propagation, yield, fruit quality (particularly anthocyanin content), and storage life. It is also beneficial for increasing the intensity of chlorophyll and the growth of roots and vegetative mass without any negative side effects. Agrostemin® consists of two groups of organic compounds: the active complex and the inhibitors. The active complex is a mixture of free amino acids and derivatives of amino acids, as well as organic acids and derivatives of organic acids. These include allantoin, tryptophan, folic acid, orcialanine, glutamic acid, allantoic acid, and adenine. The inhibitors in traces are derivatives of ABA (abscisic acid), saturated aliphatic hydrocarbons, and the cyclic inhibitor (C_8_H_29_N_3_O_7_) in trace amounts [38,39].

Crops® bioregulator is a natural product that is a concentrated liquid solution based on chitosan, natural extracts, and vitamin C. Its principal benefits are to accelerate germination and plant and root growth, promote the growth of beneficial microorganisms, strengthen the immune system of the plant, and increase yield by enhancing chlorophyll production and nutrient absorption [40,41]. Chitosan, which is the main active ingredient in Crops®, is mainly used as a plant defense booster and acts by triggering various physiological and morphological responses within the plant, thereby stimulating natural defense mechanisms and improving crop vigorousity [42].

### 2.4 Treatments and treatment combinations

The treatments consist of three rates of urea (46% N) fertilizer (at 0, 100, and 150 kg/ha), three types of bioregulators (Agrostemin 30 gm/ha, Crops® 30 mL/ha, and no bioregulator), and two varieties of sugarcane (NCo-334 and D42/58), resulting in eighteen treatment combinations (2 × 3 x 3) (Table 1). The levels of N fertilizer (Urea, 46% N) were defined based on the conventionally used rate of urea (46% N), which is 150 kg/ha (69 kg N), as described by Refs. [43,44]. Hence, the 150 kg/ha and 0 kg/ha rates of urea were used as a positive control and a negative control, respectively, for comparison. The size of each experimental plot was 43.5 m^2^ (6 furrows of 5 m in length and 1.45 m in width). The distances between adjacent plots and blocks were 1.50 and 2.90 m, respectively. Border spaces of 2.9 m and 3 m along and across replications were used. Each plot contained 150 three-budded setts, and each furrow contained 25 three-budded setts. The net plot area for data collection was 29 m^2^ (four furrows of 5 m in length and 1.45 m in width). The treatments were arranged in a combined factorial randomized complete block design (RCBD) and replicated three times.

**Table 1:** Descriptions of the treatment factors (varieties, N fertilizer rates, and bioregulators) and combinations.

Treatment	Combination of the Treatments		
	Varieties	N fertilizer rates (kg/ha urea)	Bioregulators
1	D42/58	0	No bioregulator
2	D42/58	0	Agrostemin (g/ha)
3	D42/58	0	Crops® mL/ha
4	D42/58	100	No bioregulator
5	D42/58	100	Agrostemin (g/ha)
6	D42/58	100	Crops® mL/ha
7	D42/58	150	No bioregulator
8	D42/58	150	Agrostemin (g/ha)
9	D42/58	150	Crops® mL/ha
10	NCo-334	0	No bioregulator
11	NCo-334	0	Agrostemin (g/ha)
12	NCo-334	0	Crops® mL/ha
13	NCo-334	100	No bioregulator
14	NCo-334	100	Agrostemin (g/ha)
15	NCo-334	100	Crops® mL/ha
16	NCo-334	150	No bioregulator
17	NCo-334	150	Agrostemin (g/ha)
18	NCo-334	150	Crops® mL/ha

### 2.5 Planting and treatment application procedures

The experimental field was plowed three times and prepared to a fine seedbed mechanically, and each plot was prepared manually before planting. Ten months old, healthy, three budded, and homogeneous cane setts were ready a day before planting from the initial seed cane field of the estate. Lysol solution (120 mL of Lysol in 1 L of water) was used for sterilizing the knives after each cutting of stool and chopping off one stalk to keep the setts from contamination. The setts were then wholly immersed in the solution of fungicide, Benlate/Benomyl (180 g in 200 L of water), for 1–2 min using a steel wire basket that was made for the free flow of chemical solution in the drum to protect the chopped sett from soil-borne diseases and pests after planting. The chemical solution was then drained from the drum, and the chopped cane setts were removed from the steel wire basket following treatment [43].

The treated cane setts were planted in the furrow with a 5 cm edge overlap of the sett arrangement in the pre-irrigated experimental field on March 16, 2018. The numbers of setts to be planted per 5-m furrow length were determined using the average length of setts prepared for planting by running a simple trial on the field. Accordingly, twenty-five setts, each having three buds, were planted per 5-m furrow length. At planting time, the conventional recommended rate of 300 kg/ha NPS was manually applied to all experimental plots in the furrows. On the plots treated with the Agrostemin bioregulator, Agrostemin was applied to the planted sett canes at a rate of 30 g ha^−1^ in 1000 l of water using a knapsack sprayer and then immediately covered with a 2–5 cm layer of soil. Bioregulator Crops® application was also performed by spraying with a knapsack sprayer at 20, 35, 50, and 65 days after planting at 30 mL/ha in 100 mL water. As a source of nitrogen fertilizer, urea (46%), at rates of 0, 100, and 150 kg ha^1^ (69 kg N), was applied at the age of 2 and a half months after planting. Except for nitrogen fertilizer, all agronomic management practices were carried out in accordance with the estate's recommendations.

### 2.6 Sampling and analysis of soil of the study site

Two composite soil samples were collected diagonally from five spots of the experimental site at two depths (0–30 and 31–60 cm) before planting. Uniform slices and volumes of soil were obtained in each sub-sample by the vertical insertion of an auger. The soil samples were air-dried, ground using a standard soil sample grinding machine, and allowed to pass through a 2 mm sieve. Working soil samples were obtained from each sample and analyzed for selected soil physicochemical properties at the Wonji Research and Training Laboratory and the Fincha'a Research Station Laboratory of the Sugar Corporation.

The particle size distribution was determined by the hydrometer method (differential settling within a water column) using particles less than 2 mm in diameter [45]. The soil pH and EC were measured in a 1:2.5 soil-to-water ratio using a pH meter and a conductivity meter, respectively [46]. Soil organic C and CEC were determined by the volumetric and ammonium acetate method, respectively [47]. The total N was analyzed by the Micro-Kjeldahl digestion, distillation, and titration method with sulfuric acid [46]. The available P was determined following the procedure of Olsen [48]. The available K was extracted by sodium acetate trihydrate solution, and the amount was determined using a flame photometer [49].

### 2.7 Data collection and measurements

**Sprout/germination count:** The germination count was recorded 45 days after planting upon completion of germination. The number of shoots in the middle rows was counted and converted into germination percentage.

**The total number of tillers:** A total number of tillers was recorded from each plot monthly, starting from 2 months to 4 months three-wise and then converted into a number of tillers per hectare.

**Stalk population:** Stalk population was counted at monthly intervals from crop ages of 5 months–10 months per plot and then converted to per hectare.

**The number of millable canes:** Millable canes are canes that have attained normal height and thickness at physiological maturity and are ready to be harvested for processing. A number of millable canes were counted at ten months of age in four rows of each experimental plot and then converted to a number of millable canes per hectare.

**Plant height:** The stalk length of twelve randomly selected plants were measured in cm from bottom to apices or top visible dewlap at age of 10 months.

**Cane girth:** Cane girth was measured in each plot at the top middle and bottom part of the twelve randomly selected (tagged) stalks using a vernier caliper in cm and the average was worked out.

**Single cane weight:** Twelve randomly selected stripped canes from each experimental unit were weighed together and then averaged.

**Cane yield**: Cane yield per hectare was estimated as a product of average single cane weight, and the total stalk population was counted in each experimental unit at a crop age of 10 months.

**Cane juice quality**: The juice was extracted from 12 stalk samples using a Jeffco mill for cane quality analysis. The quality parameters determined include Brix percentage (%), polarization percentage (Pol %), purity percentage (%), and recoverable sucrose percentage (%). The Brix % of the juice was measured by the Brix hydrometer in the cane juice analytical laboratory of Fincha'a Sugar Factory [50]. The Pol % of juice was measured by a polarimeter following the method described by Horne's dry lead [51]. Purity (%) was determined by the percentage of sucrose or pol in the juice based on its brix [52]. The percent recoverable sucrose was determined using the model used by Wonji Sugar Corporation Research Laboratory as described by Kassa [53] using the following formula:

Recoverablesucrose(%)={%pol−(%brix−%pol)0.52}0.75

where, 0.52 = non-sugar factor and 0.75 = cane factor.

**Sugar yield determination**: The sugar yield of the cane was determined as the product of cane yield per hectare and estimated recoverable sugar [54], using the following formula:

$$Sugar\;yield\;\left(\frac{t}{ha}\right)=\frac{Cane\;yield\;\left(\frac{t}{ha}\right)x\;ERS\;\left(\%\right)}{100}$$

where, ERS = Estimated recoverable cane sucrose.

### 2.8 Data analysis

The data were analyzed by SAS software statistical packages using analysis of variance (ANOVA). Mean comparisons were performed using the Least Significant Difference (LSD) at a 5% significance level. The Pearson correlation analysis was done using the IBM statistical package for social science (SPSS, v22) to determine the relationship among cane growth, yields, and juice quality parameters.

## 3 Results and discussion

### 3.1 Soil characteristics before planting and after harvesting

The results of soil physicochemical properties analysis before planting and after harvest (10-months aged cane) at two soil depths (0–30 and 30–60 cm) are shown in Table 2. The results indicated that the soil textures before and after planting are dominated by sand and clay fractions. Based on the soil texture determination triangle, the soils of the experimental site were sandy clay. Such soil textural class could be attributed to the mixing of surface and subsurface soils during tillage activities and subsoiling operations, and to surface runoff due to irrigation in the experimental field. The dominance of sand and clay fraction distribution in the sugarcane cultivated soils was reported [55]. From previous reports, the particle size distribution of the soils in the Fincha'a watershed ranged from sandy clay to clay, due to the effect of pedogenesis processes such as erosion, deposition, eluviation, weathering, and cultivation [36].

**Table 2:** Soil physicochemical properties of the experimental site before planting and after harvest of 10-months aged cane at two sampling depths (0–30 and 30–60 cm).

Sampling depth (cm)	Soil physicochemical properties of the experimental soil before planting										
	Soil physical properties				Soil chemical properties						
	Soil texture (%)			Textural class							
	Sand	Silt	Clay		pH	EC	OC	TN	AP	AK	CEC
0–30	49	10	44	sand clay							
30–60	38	12	52	sand clay	6.20	0.18	1.3	0.07	4.45	204	52.9
Mean	43.5	11	48	–	6.20	0.14	1.5	0.06	6.32	291	48.9
Soil physicochemical properties after harvesting a 10-months aged sample stalk											
0–30	49	10	44	Sand clay	6.05	0.05	0.95	0.02	7.85	368.00	49.00
30–60	38	12	52	Sand clay	6.14	0.07	1.00	0.05	4.05	195.00	52.90
Mean	43.5	11	48	–	6.01	0.06	0.98	0.035	5.95	281.50	50.95

EC: electrical conductivity (ds/m); OC: organic carbon (%); TN: total nitrogen content (%); AP: available phosphorus content (mg/kg soil); AK: available potassium content (mg/kg soil); CEC: cation exchange capacity (meq/100 g soil.

Higher sand contents were found in the topsoil layer (0–30 cm) followed by clay contents both before planting and after harvest. This might be attributed to the soil mixing up from tillage activities and intensive and continuous cultivation leading to compaction on the topsoil layer, decreasing the translocation of sand particles. In contrast, higher clay content (52%) in the subsurface soil layer (30–60 cm) than in the topsoil layer might indicate higher clay translocation from the topsoil layers to the bottom layer. In the two soil depths (0–30 and 30–60 cm), higher mean contents of the clay particles were obtained within both the surface and subsurface soil layers, while the lowest mean silt contents were observed in both the surface and subsurface soil layers. An increase in clay content with increasing soil depth and the lowest overall mean proportion of silt content [36]. Similarly, Tesfaye et al. [55] reported higher sand content at the surface and higher clay content at the subsurface layers of the soils, and attributed to the movement of clay in the surface layers downwards and building up in the layers below.

According to Murphy [56] classification, the soils were typically slightly acidic with a pH of 6.20 before planting at both sampling depths (0–30 and 31–60 cm). After harvesting 10-month-aged cane, the pH was slightly reduced to 6.05 and 6.14 at a sampling depth of 0–30 cm and 31–60 cm, respectively (Table 2). The soil pH of Fincha'a Sugar Estate was slightly acidic due to the removal of basic cations by excessive rainfall or the leaching of bases by percolating water [55]. Cherubin et al. [57] stated that the optimum soil pH requirement for sugarcane cultivation ranges between 6.00 and 7.00 and, hence, the pH of the study area is in the range ideal for sugarcane production.

The soil low EC values were low at both soil sampling depths before planting, which were 0.1 and 0.18 ds/m at a depth of 0–30 cm and 31–60 cm, respectively, and after harvesting of sugarcane, which were 0.05 and 0.07 ds/m at a depth of 0–30 cm and 31–60 cm, respectively (Table 2). Based on Hazelton and Murphy [58] rating for EC of soils, the EC values observed in this study were <2 ds/m, rated as low EC, designating a non-saline soil condition. The observed low EC values and its variation within the soil depths could be attributed to the high rainfall amount of the area and irrigation used for the sugarcane production, which led to the leaching away of base-forming cations from the surface and subsurface soils. Furthermore, long-term conventional sugarcane cropping practices might have resulted in lower salt content in soils. Besides, the low variation in soil EC within the soil depths indicates that the salt concentrations found in these soils were leached consistently throughout the soil profiles. Similarly, Siqueira et al. [59] reported differences in soil EC in soil depth layers and attributed it to the soil water contents, as water contents become more stable in the deep layers. As a result, the higher EC values were observed in the subsurface soils than in the surface soils, both before and after planting, and the EC before planting decreased with an increase in soil depth. Dengia and Lantinga [20] reported that soil EC at the subsurface depth (30–60 cm) was higher than at the surface depth (0–30 cm) and attributed this to the leaching of salts from the top layer of the soil to lower soil horizon due to excessive irrigation and rainfall. In contrast, Kitila et al. [36] indicated that the soil EC decreased gradually with an increase in the soil depth.

The percent OC of the soils was moderate at a sampling depth of 0–30 cm and low at a sampling depth of 30–60 cm before planting (Table 2). After ten months of aging, the percent organic carbon was low at both sampling depths based on the rating of Tadesse et al. [60]. The low organic carbon contents of the soils after planting could be attributed to the high rate of mineralization as continuous cultivation increases soil aeration, thereby improving breakdown of soil organic matter, and the plant removes most of the soil organic matter found in the soils. The soil OC and OM contents were higher at the topsoil layer than at the subsurface before planting, while it is lower in the topsoil than in the soil below after harvesting. These higher soil OC and OM contents at the topsoil layer before planting might be attributed to the higher soil organic matter buildup due to high cane roots and above-ground biomass inputs. The lower soil OC and OM contents in the topsoil layer after harvest, on the other hand, could be attributed to cultivation practices that increased soil aeration and enhanced decomposition, as well as harvesting, which removes the majority of the soil's OM. Kitila et al. [36] also stated that the lower soil OC content was caused by a greater rate of OM oxidation as a result of continuous irrigation and a lower amount of soil OM restored to the soil system owing to losses due to water erosion and removal of green materials. Tena et al. [1] and Wakgari [61] also obtained low organic carbon content due to low levels of organic matter accumulation and continuous tillage practices during land preparation under a sole and intensive cane cropping system.

The total N content of the soil was 0.05% and 0.07% for the sampling depths of 0–30 cm and 31–60 cm, respectively, before planting the cane (Table 2). According to Tadesse et al. [60] rating, these total N contents are rated as very low and low for the sampling depths of 0–30 cm and 31–60 cm, respectively. However, the total N content of the soil after harvesting ten-month-old cane were 0.02% and 0.05% for the depths of 0–30 cm and 31–60 cm, respectively, which are rated as very low. The very low total N contents and its reduction after harvest at both depths indicate that most of the N found in the soils was removed with harvest, as sugarcane is a heavy feeder and takes up the N primarily, as also indicated by Tena et al. (2016), requiring supplemental N for sustainable production. The differences in the total N contents among soil depths could be attributed to the differences in soil OM contents between the two soil depths [55]. The lower distribution of total N contents at 0–30 cm depth than at 30–60 cm could be because sugarcane is a shallow-rooted plant and removes minimal soil nutrients from the deeper soil depth. Dengia and Lantinga [20] and Kitila et al. [36] also found similar trends in the total N content of the soils where sugarcane is grown.

The soil of the experimental field had medium available P content (8.18 mg/kg soil) at depth of 0–30 cm and low content of available P (4.45 mg/kg soil) at depth of 30–60 cm before planting, based on the rating of Olsen [48]. After harvesting 10-month aged cane, the available P contents of the soil was medium (7.85 mg/kg soil) at a sampling depth of 0–30 cm and low (4.05 mg/kg soil) at a sampling depth of 30–60 cm (Table 2). This indicates that the soil P has been subjected to long periods of P adsorption by the soils and extraction by the crops whose residues were not recycled into the soil, as also stated by Balemi and Negisho [62]. Higher levels of available P were observed at the topsoil layer both before planting (8.18 mg/kg soil) and after harvest (7.85 mg/kg soil), which might be related to the application of P fertilizers to the surface soils for fertility improvement. Besides, the decrease in available soil P level with an increase in soil depth could be attributed to an increase in the clay content of the soil with depth, which caused the fixation of P. Tesfaye et al. [55] found that the amount of available soil P decreased with soil depth, while more sugarcane residues and better biological activities in the topsoil layer increased the amount of available P in the topsoil layer. These authors also indicated that sugarcane can uptake P from the topsoil and that soil P in the subsoil decreases due to fixation with clays.

The available K contents were within the high ranges, which were 377 mg/kg of soil at a depth of 0–30 cm and 204 mg/kg of soil at a depth of 30–60 cm before planting, while it was 368 mg/kg soil and 195 mg/kg of soil after harvest at a depth of 0–30 cm and 30–60 cm, respectively (Table 2). Similar to P, higher available K was observed at the topsoil layer both before planting (377 mg/kg soil) and after harvest (368 mg/kg soil), which might be correlated to the application of K fertilizers to the soils. Previous studies have also indicated that higher K levels in the soils could be attributed to parent materials, weathering, land-use types, fertilizer types, leaching rates, crop remains, and litterfall [1,20,63]. Furthermore, Dengia and Lantinga [20] stated that the critical value of available K ranges from 78 to 125 mg/kg and hence, the available K in the study soil is sufficient for sugarcane production.

The CEC of the soil before planting was 44.9 and 52.9 meq/100 g of soil for the sampling depths of 0–30 cm and 30–60 cm, respectively, while the CEC of the soil after ten months of planting was 49.00 and 52.90 meq/100 g of soil for the sampling depths of 0–30 cm and 31–60 cm, respectively (Table 2). It can be noted that the CEC of the soil increased following the sugarcane planting and nitrogen fertilizer application. According to Tiruneh et al. [63], a significant change and higher CEC indicated that the soils of the study area have a high capacity to retain nutrients against leaching losses. The highest CEC values recorded at both soil depths may be due to differences in soil organic material and quantity and type of clay, which adsorb and retain positive cations through electrostatic force. Studies have revealed that the CEC of sugarcane-friendly soils was typically greater than 15 meq/100 g of soil [50,64] and as a result, the soils of the experimental site can generally be considered fertile for sugarcane production.

### 3.2 Effects of treatments on sugarcane growth, yield, and quality parameters

#### 3.2.1 Sugarcane growth

**Fig. 1:**
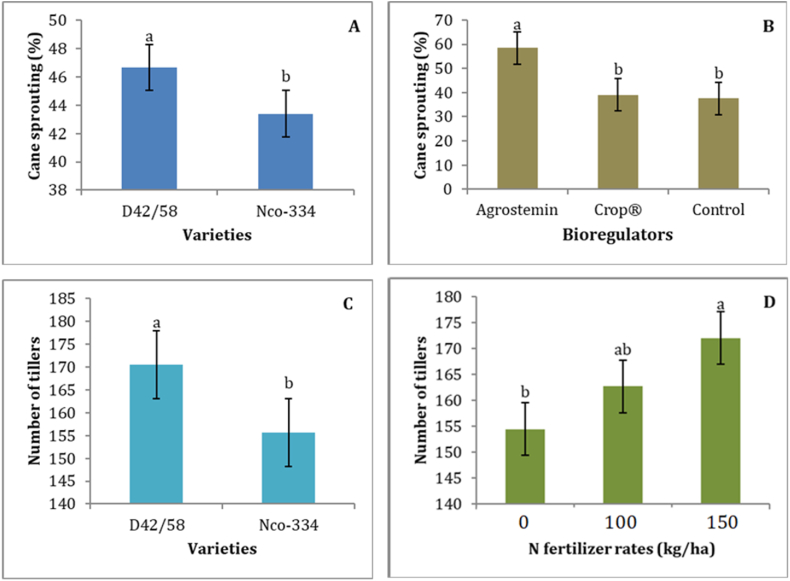
Cane sprouting as influenced by varieties (Figure A) and bioregulators (Figure B) and the number of tillers as influenced by varieties (Figure C) and N fertilizer rates (Figure D). Means with the same letter (above the bars of each figure) are not significantly different by the LSD test at the 5% level of significance. The error lines represent the standard error of the mean.

The results of the study revealed that sprouting, tillering, plant height, number of millable canes, cane thickness/girth, and cane weight were significantly (P < 0.05) affected by sugarcane varieties and bioregulators (Table 3). The D42/58 variety recorded the highest mean of sprouting (46.64%), whereas the NCo-334 variety recorded the lowest mean of sprouting (43.39%) (Fig. 1A). The difference in sprouting among sugarcane varieties clearly indicates that sprouting is greatly influenced by varietal characters, which could be mainly attributed to the difference in the genetic makeup of the varieties. The results are supported by previous reports by Refs. [1,65,66]. Further, Burayu et al. [19] in the report of the same study location, revealed that the difference in overall growth, yield, and yield-contributing attributes among sugarcane varieties was strongly dependent on cane genotypes.

**Table 3:** Analysis of variance for sugarcane growth, yield, and juice quality parameters, and sugar yield as affected by a variety, N fertilizer rates, and bioregulators at Fincha'a Sugar Estate.

Treatments	Mean squares of sugarcane growth, yield, and juice quality parameters, and sugar yield												
	ST	NT	PH	CG	MC	SP	CW	CY	BX	PL	PR	SC	SY
Vr	142.43**	2955.56**	12830.24**	138.46**	2.6 × 10^9^**	2.1 × 10^9^**	0.64**	4181.28*	0.30**	39.48**	689.80**	8.59**	2.77**
NF	11.93^ns^	1393.42**	550.07^ns^	4.25^ns^	1.93 × 10^8ns^	2.2 × 10^7ns^	0.08*	2543.35*	18.64**	30.03**	194.00**	30.03**	0.06^ns^
VrxNF	36.07^ns^	85.97^ns^	7.64^ns^	5.05^ns^	1.05 × 10^8ns^	1.18 × 10^8^*	0.07^ns^	2368.00*	0.16**	2.96**	14.34**	0.22^ns^	0.30^ns^
Br	2455.40^ns^	153.21^ns^	17890.03**	0.66^ns^	3.1 × 10^7 ns^	4.02 × 10^8^**	0.04*	568.60^ns^	0.91**	3.39**	34.29**	0.01^ns^	0.97**
VrxBr	34.30^ns^	55.50^ns^	1321.26^ns^	5.86^ns^	1.89 × 10^8ns^	4.62 × 10^8^**	0.04^ns^	1608.30^ns^	0.84**	1.40**	9.87**	0.11^ns^	0.40**
NFxBr	18.98^ns^	506.96^ns^	951.13^ns^	1.46^ns^	1.9 × 10^8 ns^	1.28 × 10^8^*	0.02^ns^	990.06^ns^	0.20**	1.43**	11.72**	0.09^ns^	0.05^ns^
VrxNFxBr	10.27^ns^	509.13^ns^	700.84^ns^	0.58^ns^	1.81 × 10^8ns^	1.52 × 10^8^**	0.014^ns^	171.20^ns^	0.15**	0.45**	12.01**	0.04^ns^	0.05^ns^
MSE	17.14	202.64	548.00	2.24	1.17 × 10^8^	3.44 × 10^7^	0.02	622	0.0018	2.71	1.01	0.10	0.06
CV (%)	9.20	8.73	9.22	6.40	7.76	6.80	16.22	18.86	0.28	2.05	1.23	3.40	2.54

Where, * indicates that the mean squares are significant at the 5% level of significance; ** indicates that the mean squares are highly significant at the 5% level of significance; ^ns^ indicates that the mean squares are non-significant at 5% level of significance; Vr = variety; NF = N fertilizer rates; Br = bioregulators; ST = sprouting (%); NT = number of tillering; PH = plant height (cm); CG = cane girth (mm); MC = number of millable canes; SP = stalk population; CW = cane weight (kg); CY = cane yield (tons/ha); BX = Brix (%); PL = Pol (%); PR = purity (%); SC = sucrose content (%); and SY = sugar yield (tons/ha).

Treating the seed cane with the Agrostemin bioregulator resulted in the highest mean of sprouting (58.47%), while the control resulted in the lowest sprouting mean (37.50%), which was statistically at par with treatment with Crops® (39.07%) (Fig. 1B). Hence, treating the cane sett with bioregulators improved sprouting, probably through stimulating the initiation of bud sprouting and maintaining the optimal environment, such as optimum moisture levels. In contrast, delayed and poor sprouting with untreated setts suggested a difficulty in the initiation of sprouting and the respective initiation of shoots at an early stage, as these traits rely on adequate moisture provided with the treatment. Plant growth bioregulators are applied to sugarcane setts to improve germination, growth, juice quality, and sugar yields [67]. da Silva et al. [68] also observed that sugarcane genotypes responded differently to biostimulants in the absence or presence of fertilizers. In the same way, Patel and Chaudhary [69] explained that bioregulators could be used to stimulate and cause sprouting at lower nodes, which led to a better plant establishment and required fewer planting materials.

Tillering capacity and tiller population are the main important crop variables that are used to estimate the final cane population and sucrose yields [70]. In this study, both variety and rate of nitrogen fertilizer had a significant (P < 0.05) effect on the number of tillers (Table 3). The D42/58 variety recorded the highest (170.43) and the NCo-334 variety recorded the lowest (155.64) total mean number of tillers (Fig. 1C). This is inconsistent with Getaneh et al. [37], who showed that the NCo-334 variety has high tillering potential at Fincha'a Sugar Estate. Previous studies were also revealed the difference in tiller development among sugarcane varieties mainly attributing to the existence of considerable genotypic differences as tillering ability is a genetically governed trait [13,71,72]. The mean comparison of N fertilizer application showed that the application of 150 kg/ha recorded the highest mean number of tillers (172.02), while the control recorded the lowest mean number of tillers (154.44), followed by the application of 100 kg/ha (162.64) (Fig. 1D). In both sugarcane varieties, the number of tillers per plant increased with increasing nitrogen application, which was consistent with the findings of Ashraf et al. (2008). Studies have also shown that nitrogen is required more for sugarcane vegetative growth such as tillering, stalk growth, internode formation and elongation, an increase in cane girth and weight, and root growth [24,25,54,70,73].

**Fig. 2:**
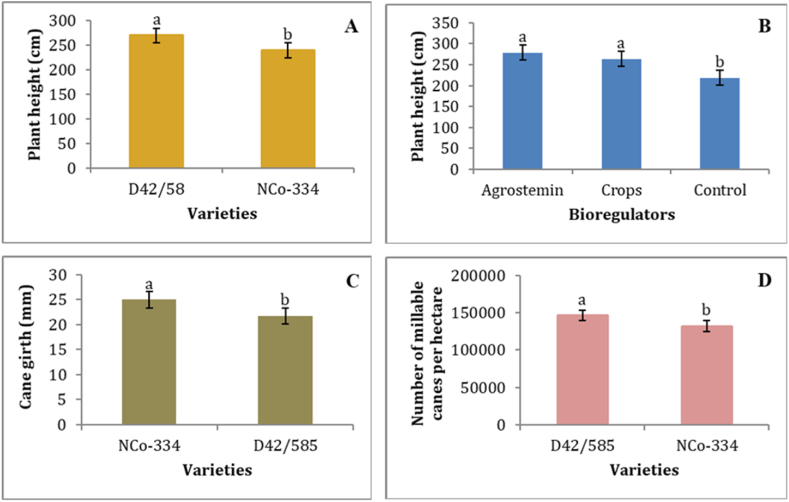
The effect of sugarcane varieties on the plant height (Fig. 2A), cane girth (Fig. 2C) and number of millable canes per hectare (Fig. 2D), and the effect of bioregulators on the plant height (Fig. 2B). Means with the same letter (above the bars of each figure) are not significantly different by the LSD test at the 5% level of significance. The error lines represent the standard error of the mean.

The results this study confirmed that variety and bioregulators significantly (P < 0.05) affected sugarcane plant height (Table 3). However, nitrogen fertilizer rates, as the main factor, and the interactions of the three factors had no significant (P < 0.05) effect on the plant height. The D42/58 variety gave significantly the highest mean plant height (269.23 cm), while the NCo-334 variety gave the lowest mean plant height (238.40 cm) (Fig. 2A). These findings suggest that varietal characteristics have a strong influence on plant height and associated traits such as internode and node elongation and length. Furthermore, the differences in the performances of the varieties could be attributed to differences in their ability to respond to the applied treatments, which affects their ability to grow under given conditions. Similarly, Getaneh et al. [37] reported significant differences in the height of cane among different sugarcane varieties at Fincha'a Sugar Estate. The height of cane can also be affected by varieties and different soil types as described by Tadesse [74]. The application of bioregulators (Agrostemin and Crops®) also resulted in a higher mean of plant height than the control treatment (Fig. 2B). Treating the seed cane with the Agrostemin bioregulator resulted in the highest mean of plant height (279.06 cm). Ayele et al. [75] reported that the application of Agrostemin bioregulator produced significantly higher cane height than the treatment that didn't receive bioregulator. Patel and Chaudhary [69] also stated that plant bioregulators have a beneficial effect on cane length through stimulated cane growth and increased stem elongation.

Cane girth was significantly affected by varieties, with the NCo-334 variety having a significantly thicker cane girth (25 mm) than the D42/58 variety (21.77 mm), which could be attributed primarily to genotypic variation (Fig. 2C). The differences in single cane girth could be attributed to the inherent differences in the genotypic makeup of the sugarcane varieties. Moreover, the NCo-334 variety had lower spouting and tillering capacity than the D42/58 variety, thus it was less influenced by competition for light, moisture, and nutrients, resulting in a thicker cane girth, and hence cane thickness is a variety trait. Significant variations for growth attributes such as cane thickness, cane length, diameter of millable cane, and millable stalk population among the sugarcane varieties were also reported by Refs. [13,29,76]. In this study, nitrogen fertilizer application rates had no significant effect on cane girth. Likewise, the findings of [64,77] revealed the absence of significant variation in single cane girth due to the application of nitrogen fertilizer.

The number of millable canes was also significantly influenced by the varieties (Fig. 2D). The rates of nitrogen fertilizer and bioregulators and the interaction of the three factors (variety, fertilizer, and bioregulator) did not have a significant (P < 0.05) effect on the number of millable canes (Table 3). This is in line with Bokhtiar et al. [78] and Kamboj [82], who found no significant difference in the number of millable canes as the dose of nitrogen fertilizer increased. The mean comparison for varieties showed that the D42/58 variety produced the higher mean number of millable canes (146,028), whereas the NCo-334 variety produced a lower (132,146) number of millable canes per hectare (Fig. 2D). The D42/58 variety produced higher millable canes due to its maximum tillering capacity, which was supported by the strong positive and highly significant correlation between the number of millable canes and the number of tillers (Table 11). This finding agrees with the results [29,79,80], who reported a considerable variety of differences in the number of millable canes. A study by Alemu et al. [81] also revealed that sugarcane varieties with good tillering capacity produced the highest number of millable canes.

Variety, bioregulators, and their interaction effects, as well as the interaction of variety, bioregulators, and N fertilizer application, significantly affected the cane stalk population (Table 3). The maximum mean values of the stalk population (107126) were obtained from the combination of D42/58 variety, 100 kg N/ha, and Agrostemin treatment, which was at par with the combination of D42/58 variety, 0 kg N/ha, and Agrostemin treatment. However, the minimum mean values of the stalk population were recorded from combined treatments NCo-334, 0 kg N/ha, and no bioregulator; NCo-334, 150 kg N/ha, and no bioregulator; NCo-334, 150 kg N/ha, and Agrostemin; and NCo-334, 150 kg N/ha, and Crops®’ (Table 4). The D42/58 variety gave the highest stalk population, which could be due to its higher tillering capacity, resulting in the production of a higher number of stalks. As a result, the number of tillers could be an important parameter that could be used to estimate the final stalk population and sucrose yields in sugarcane. Ayele et al. [75] also revealed that the application of urea and Agrostemin produced a significantly higher number of tillers, while the treatment that didn't receive either urea or a bioregulator produced the lowest number of tillers. A previous report by da Silva et al. [68] also showed that bioregulators can promote axillary bud break and improve initial shoot and foliage numbers in sugarcane species.

**Table 4:** The interaction effect of varieties, N fertilizer rates, and bioregulators on stalk population.

				Varieties		
		D42/58			NCo-334	
**N fertilizer rates (kg/ha)**	0	100	150	0	100	150
**Bioregulators**						
Agrostemin	107126^a^	107126^a^	95709^abc^	78850^cd^	82605^cd^	76169^d^
Crops®	79847^cd^	83142^cd^	101839^ab^	83678^cd^	79923^cd^	76169^d^
Control	82452^cd^	84904^bcd^	87433^bcd^	77241^d^	84751^bcd^	77241^d^

Means with the same letter within the same columns are not significantly different by the LSD test at the 5% level of significance.

#### 3.2.2 Sugarcane yield components and yield

**Fig. 3:**
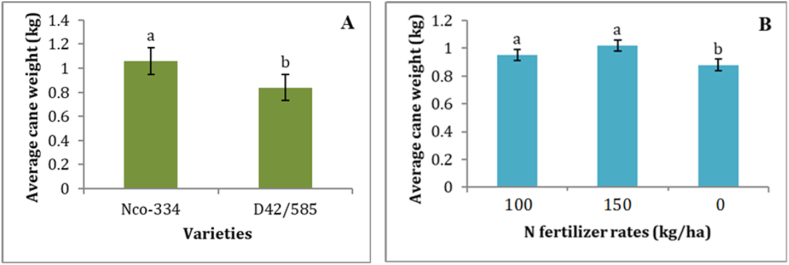
The influence of varieties (Fig. 3A) and N fertilizer rates (Fig. 3B) on average cane weight. Means with the same letter (above the bars of each figure) are not significantly different by the LSD test at the 5% level of significance. The error lines represent the standard error of the mean.

The study revealed that the weight of a single cane was significantly affected by variety, N fertilizer rate, and bioregulators (Table 3). The NCo-334 sugarcane variety produced significantly more mean single cane weight (1.06 kg/single cane) than the D42/58 variety (Fig. 3A). Previous findings reported differences among sugarcane genotypes in several millable canes, cane thickness, and cane weight [66,78,79]. Although cane weight is a function of cane thickness, stalk height, and stalk density [70], the higher cane weight in NCo-334 variety could be mainly attributed to the higher cane girth (25 mm) of this variety. The reduction of cane weight in the D42/58 variety, which had the highest number of tillers and stalk population, could be attributed to the induced competition for resources (light, moisture, nutrients, etc.) and the presence of insubstantial canes after the competition. Similarly, Legesse et al. [70] reported that cane girth plays a dominant role in improving cane yield per unit area due to its effect on increasing stalk weight.

Variety and the sole application of bioregulators significantly (P < 0.05) influenced the cane weight (Table 3). This is in agreement with the finding of Ayele et al. [75], who reported that the sole application of Agrostemin and Crops® significantly increased cane weight by 7.37 and 9.50%, respectively, over the control (no application of a bioregulator). Different rates of nitrogen fertilizer application also significantly (P < 0.05) influenced the cane weight (Table 3). The higher dosage of nitrogen fertilizer (150 kg urea/ha) resulted in a higher cane weight with 15.90% increase than the control treatment although it is on par with the application of 100 kg urea/ha (Fig. 3B). An increase in the cane weight was noted with an increase in the application of nitrogen fertilizer, and this could be attributed to the improvement in stalk growth, internode elongation, cane girth, and root growth. Several studies have revealed that the application of nitrogen fertilizers could improve cane growth, weight, and sugar yield per unit area [25,43,73,82].

Analysis of variance showed that cane yield was significantly affected by the two main factors, i.e., variety and rate of fertilizer, and the interaction of these factors (Table 3). The maximum cane yield mean of 153.34 tons/ha was obtained from the D42/58 variety with application of 100 kg urea/ha, which was not significantly different from the control and 150 kg urea per hectare application (Table 5). This cane yield is 20.55% higher than the cane yield obtained from the same variety that received control treatment i.e., application of 0 kg urea/ha. The lower mean of cane yield (111 tons/ha) was recorded for the NCo-334 variety with the application of 100 kg N/ha, but the value was statistically not different from the same variety with control treatment (no application of urea) (113.76 tons/ha) (Table 5). The higher cane yield of the D42/58 variety might be attributed to early cane germination and a higher number of tillers, stalk population, and number of millable canes. This could be supported by the positive relationship between cane yield and parameters such as number of tillers, plant height, number of millable canes, and stalk population (Table 11). These results conform to Birhanie et al. [54], who reported that cane yield is a result of approximately 70% of millable canes, 27% of cane length, and 3% of cane girth. Similarly (Alemu et al., 2018), showed that cane yield depends on components such as the number of tillers and millable stalks and plant height. Nitrogen fertilizer application could also affect cane yield by improving the emergence rate, tillering capacity, cane length and diameter, millable canes and cane weight [25]. Numerous studies by have also revealed that nitrogen fertilizer application in sugarcane significantly increased crop stand as well as cane yield and quality [24,43,70,73]. Besides, cane yield could also be significantly affected by varieties of cane [37,72,74,80].

**Table 5:** The interaction effect of sugarcane varieties and N fertilizer rates on cane yield.

N fertilizer rates (kg/ha)	Varieties	
	D42/58	NCo-334
0	127.20^a^	113.76^b^
100	153.34^a^	111.00^b^
150	142.76^a^	145.72^a^

Means with the same letter within the same columns are not significantly different by the LSD test at the 5% level of significance.

#### 3.2.3 Cane juice quality and sugar yield

The cane juice quality parameters were significantly affected by the three main factors: sugarcane variety, rate of nitrogen fertilizer, and bioregulator, as well as their combinations, except for sucrose percent, which was only significantly affected by variety and rate of fertilizer application (Table 3). The interaction of the three main factors also showed a significant (P < 0.05) effect on the Brix percentage (%), Pol percentage (%), and purity percentage (%) parameters. The maximum mean value of Brix % (16.91%) was recorded from the combination of NCo-334 variety, 150 kg N/ha, and Crops®, while the minimum mean value of Brix % was obtained from plots treated with a combination of NCo-334 variety, 0 kg N/ha, and Crops® (Table 6). This indicates an increment in Brix % as rates of nitrogen increased, while treating the NCo-334 variety with 150 kg N/ha and Crops® is the optimum combination to get a higher brix % in sugarcane juice. The result of this study was supported by the finding of [23,25,66], who revealed that a difference in juice Brix % was observed among different varieties. Ayele et al. [75] also reported increased Brix % and hastened cane maturity due to bioregulators such as Agrostemin and Crops®. According to Refs. [25,73], increasing the rate of nitrogen levels 90 days after planting (DAP) significantly increased stalk height, diameter, brix %, sucrose %, purity %, sugar recovery %, number of millable canes, cane yield, sugar yields, and impurities %.

**Table 6:** The interaction effect of sugarcane varieties, N fertilizer rates, and bioregulators on Brix percentage.

	Varieties					
	D42/58			NCo-334		
**N fertilizer rates (kg/ha)**	0	100	150	0	100	150
**Bioregulators**						
Agrostemin	14.77^k^	15.78^f^	16.61^b^	14.25^l^	15.21^i^	16.3^c^
Crops®	14.20^l^	15.23^i^	16.11^d^	14.02^m^	15.64^gh^	16.91^a^
Control	14.14^lm^	15.53^h^	15.97^e^	14.01^m^	14.97^j^	15.7f^g^

Means with the same letter within the same columns are not significantly different by the LSD test at the 5% level of significance.

Pol percentage was also highly significantly (P < 0.05) influenced by the main factors: variety, rates of nitrogen fertilizer, and bioregulators, as well as their combinations (Table 3). The maximum Pol % (15.81%) was recorded from the treatment combination of D42/58 variety, 100 kg N/ha, and Agrostemin, while the lowest Pol % (10.91%) was obtained from the combination of NCo-334 variety, 0 kg N/ha, and no bioregulator (Table 7). The Pol % recorded from the combination of D42/58 variety, 100 kg N/ha, and Agrostemin was higher by 4.9% than that obtained from the combination of NCo-334 variety, 0 kg N/ha, and no bioregulator. This suggests that using 100 kg N/ha with Agrostemin on D42/58 variety is more beneficial than using other treatment combinations on the counterpart variety, NCo-334. The difference in the response and quality parameters of sugarcane juice among the varieties could be due to the genetic difference among the varieties [19,23,74]. A report by Ayele et al. [75] also reported that canes that received the bioregulator and nitrogen fertilizer were superior in quality parameters to the canes that didn't receive either the bioregulator or nitrogen fertilizer.

**Table 7:** The interaction effect of varieties, N fertilizer rates, and bioregulators on Pol percentage.

	Varieties					
	D42/58			NCo-334		
**N fertilizer rates (kg/ha)**	0	100	150	0	100	150
**Bioregulators**						
Agrostemin	13.98^efgh^	15.81^a^	15.78^ba^	11.61^kl^	12.82^ij^	14.46^def^
Crops	13.45^ghi^	15.64^ab^	15.50^abc^	11.10^l^	13.88^efgh^	14.17^defg^
Control	13.15^hij^	13.64^efghi^	14.68^cde^	10.91^l^	12.32^jk^	14.92^bcd^

Means with the same letter within the same columns are not significantly different by the LSD test at the 5% level of significance.

Purity percentage was also significantly (P < 0.05) affected by the main factors (variety, N fertilizer rate, and bioregulator), as well as their combinations (Table 3). The combined use of D42/58 variety 150 kg N/ha, and Agrostemin yielded the highest purity percentage (90%), while the combination of NCo-334 variety, 0 kg N/ha, and no bioregulator yielded the lowest purity percentage (76%) (Table 8). Tadesse [74] stated that different sugarcane varieties have different qualities of juice accumulation and purity. Besides, Zeng [25] also reported that purity % in cane juice tended to increase with an increase in the application of nitrogen fertilizer levels.

**Table 8:** The interaction effect of varieties, N fertilizer rates, and bioregulators on purity percentage.

	Varieties					
	D42/58			NCo-334		
**N fertilizer rates (kg/ha)**	0	100	150	0	100	150
**Bioregulators**						
Agrostemin	82.10^bc^	89.00^a^	90.00^a^	80.43^bcd^	79.50^cd^	81.50^bc^
Crops	81.50^bc^	87.00^a^	89.50^a^	71.50^f^	80.00^cd^	80.00^cd^
Control	80.17^cd^	83.50^b^	89.00^a^	76.00^e^	78.00^ed^	80.50^bcd^

Means with the same letter within the same columns are not significantly different by the LSD test at the 5% level of significance.

The results revealed that the two main factors, variety and N fertilizer rate, had a significant (P < 0.05) effect on sucrose percent (%) (Table 3). The D42/58 variety recorded the highest mean sucrose % (9.60%), while the NCo-334 variety recorded the lowest mean sucrose % (8.80%) (Table 9). The result follows the finding of Tadesse [74], who indicated that sucrose content in cane was affected consistently only by varieties across all soils and sugar estates in Ethiopia. The highest sucrose % (10.63%) was recorded from the application of 150 kg N/ha, whereas the lowest sucrose % (8.14%) was recorded from the control treatment (Table 10). As a result, the highest rate of nitrogen application resulted in high-quality cane juice and a high sucrose %. According to Srivastava et al. [83], the use of nitrogen fertilizers improved the purity and sucrose content of juice.

**Table 9:** The mean values of sucrose percentage of sugarcane as influenced by varieties and N fertilizer rates.

Varieties	Mean	N fertilizer rates (kg/ha)	Mean
D42/58	9.60^a^	0	8.14^c^
Nco-334	8.80^b^	100	8.81^b^
		150	10.63^a^

Means with the same letter within the same columns are not significantly different by the LSD test at the 5% level of significance.

**Table 10:** The interaction effect of sugarcane varieties and bioregulators on sugar yield.

Bioregulators	Varieties	
	D42/58	NCo-334
Agrostemin	10.25^a^	10.09^a^
Crop®	10.06^a^	9.60^b^
Control	10.10^a^	9.36^b^

Means with the same letter within the same columns are not significantly different by the LSD test at the 5% level of significance.

In the present study, sugar yield was significantly affected by variety, bioregulator, and the combination of both varieties and bioregulators (Table 3). The maximum sugar yield (10.25 tons/ha) was obtained from D42/58 variety treated with Agrostemin, while the lowest sugar yield (9.36 tons/ha) was recorded from NCo334 with no bioregulators (Table 10). The higher sugar yield of the D42/58 variety might result from higher cane germination, tillers, plant height, stalk population, millable canes, and cane yield, as revealed by the positive and significant (P 0.05) correlation of sugar yield with most parameters (Table 11). The use of 150 kg urea per ha and the Agrostemin bioregulator also showed a prominent effect on the improved sugar yield in both varieties. This result is in line with the finding of Alemu et al. [81], who revealed cane growth, yield components, and yield all have a significant impact on sugar yield. Besides, Ayele et al. [75] confirmed that the application of Agrostemin resulted in a 45.0% increase in sugar yield. Patel and Chaudhary [69] also found that bioregulators can be used to increase the accumulation of sugar in the stalk of a mature cane plant and the amount of sugar yield produced. Further, Zeng [25] also delineated that the application of 150 kg urea/ha produced the best cane quality and the highest sugar yield.

**Table 11:** Correlations analysis among sugarcane productivity and sugar yield parameters.

	ST	NT	PH	CG	MC	SP	CW	CY	BX	PL	PR	SC	SY
**ST**	1.00												
**NT**	−0.24	1.00											
**PH**	0.81*	0.32	1.00										
**CG**	−0.14	0.90**	0.46	1.00									
**MC**	−0.17	0.91**	0.43	0.99**	1.00								
**SP**	0.00	0.81*	0.58	0.98**	0.97**	1.00							
**CW**	−0.12	0.91**	0.47	0.99**	0.99**	0.96**	1.00						
**CY**	−0.35	0.78*	0.13	0.79*	0.78*	0.70	0.83*	1.00					
**BX**	−0.15	0.23	−0.24	−0.16	−0.15	−0.28	−0.10	0.24	1.00				
**PL**	−0.18	0.94**	0.28	0.77*	0.77*	0.67	0.81*	0.84*	0.50	1.00			
**PR**	−0.14	0.97**	0.44	0.97**	0.97**	0.91**	0.98**	0.84*	0.09	0.91**	1.00		
**SC**	−0.22	0.50	−0.13	0.13	0.14	0.00	0.19	0.47	0.96**	0.73	0.37	1.00	
**SY**	0.19	0.77*	0.72	0.94**	0.92**	0.97**	0.94**	0.68	−0.26	0.66	0.89**	0.00	1.00

Where, * indicates that correlation is significant at the 5% level of significance; ** indicates that correlation is significant at the 1% level of significance; ST = sprouting (%); NT = number of tillers; PH = plant height (cm); CG = cane girth (mm); MC = number of millable canes; SP = stalk population; CW = cane weight (kg); CY = cane yield (tons/ha); BX = Brix (%); PL = Pol (%); PR = purity (%); SC = sucrose content (%); and SY = sugar yield (tons/ha). The color intensities code for the strength of the correlations: light red rectangles indicate negative correlations, white rectangles indicate no correlations, and deep green rectangles indicate positive correlations.

### 3.3 Correlation among sugarcane productivity and sugar yield parameters

A correlation analysis was carried out between the different pairs of cane growth and yield, juice quality, and sugar yield parameters to determine the relationship among the various parameters studied (Table 11). The correlation analysis revealed that there was a positive correlation among most growth, yield, juice quality, and sugar yield parameters of sugarcane. The results of the correlation analysis showed that the number of millable canes had a positive correlation with all the cane growth, yields, and juice quality parameters except with cane sprouting and Brix (%), which showed a negative correlation. The relationship between the number of millable canes and the number of tillers, cane girth, stalk population, cane weight, purity (%), and sugar yield was highly significant (P < 0.05), whereas the relationship between the number of millable canes and the cane yield and Pol (%) was positive and significant (P < 0.05). Tena et al. [1] also reported a positive and significant correlation between millable cane number and cane diameter (girth), cane weight, cane yield, Pol (%), and sugar yield.

In this study, the number of tillers and cane yield had a positive correlation with all the cane growth, yields, and juice quality parameters except with cane sprouting, where a negative correlation was observed. The number of tillers had a highly significant (P < 0.05) correlation with cane girth, number of millable canes, cane weight, Pol (%), and purity (%), as well as a significant (P < 0.05) correlation with stalk population, cane yield, and sugar yield. This indicates that tillering capacity and tiller population are important parameters that determine the final cane yield, juice quality, and sugar yield. Similarly, Legesse et al. [70] also stated that tiller population is the most important factor that determines the overall crop stand and ultimately affects the cane population, cane yield, and sucrose yield. A significant correlation (P < 0.05) was also observed between cane yield and number of tillers, cane girth, number of millable canes, cane weight, pol (%), and purity (%) (Table 11). From these results, it can be noted that the number of tillers, cane girth, number of millable canes, and cane weight can be used as reliable selection criteria to improve sugarcane yield. These results are in agreement with [1,84] for cane girth, number of millable canes, cane weight, and sugar yield. A study by Tena et al. [1] further confirmed that single cane weight and number of millable canes were the major contributors to cane yield.

The correlation analysis also elucidated that the cane juice quality parameters (Pol and purity) had a positive correlation with all the cane growth and yield parameters except with cane sprouting. This indicates that improvements in cane growth and yield parameters would simultaneously result in an improvement of cane juice quality and, hence, sugar yield. Sugar yield also had a positive correlation with all parameters except brix (%) and sucrose content (%), which showed a negative correlation and no correlation, respectively. Sugar yield had a positive and highly significant (P < 0.05) correlation with cane girth, number of millable canes, stalk population, cane weight, and purity (%), as well as a positive and significant (P < 0.05) correlation with the number of tillers. Based on the magnitude of the correlation coefficient values, therefore, it can be noted that cane growth and yield parameters are much more important than quality parameters in determining the sugar yield. The same result has been reported by Tena Gashaw et al. [85], who revealed that cane yield is much more important than sucrose percent in determining the sugar yield.

## 4 Conclusions

Sugarcane productivity and sugar yield can be improved by selecting the most productive sugarcane variety and adopting proper management strategies. The present study evaluated sugarcane varieties, nitrogen fertilizer rates, and plant bioregulators for improved sugarcane production, juice quality, and sugar yield. The study revealed that all growth, yield, and juice quality parameters of the cane were significantly affected by the variety. The cane juice quality parameters were highly and significantly affected by the variety, N fertilizer rate, and bioregulator, and their interactions. Sugarcane variety D42/58 outperformed the NCo-334 variety in all growth, yield, and juice quality parameters except cane thickness and weight. Besides, the use of the Agrostemin bioregulator yielded the highest sugarcane performance, juice quality and sugar yield. The results of the correlation analysis revealed that the majority of sugarcane growth, yield, juice quality, and sugar yield parameters were positively correlated. Hence, these results could help improve sugarcane productivity and sugar yield by using strategies like high-performing varieties, optimal fertilizer rates, and plant bioregulators. Furthermore, the results suggested selecting and combining the D42/58 variety with 100 kg N/ha and/or Agrostemin bioregulator to maximize sugarcane productivity, sugar yield, and sugar quality. In general, selecting the right sugarcane variety and utilizing the optimal N fertilizer rate and plant bioregulator are key to improving sugarcane productivity and sugar yield. However, further studies across various agroecologies and seasons, with the inclusion of additional sugarcane varieties, are recommended to confirm the results.

## Author contribution statement

Belete Desalegn; Hirpa Legesse: Conceived and designed the experiments; Performed the experiments; Analyzed and interpreted the data; Contributed reagents, materials, analysis tools or data; Wrote the paper.

Erana Kebede; Tarekegn Fite: Analyzed and interpreted the data; Contributed reagents, materials, analysis tools or data; Wrote the paper.

## Data availability statement

Data included in article/supplementary material/referenced in article.

## Additional information

No additional information is available for this paper.

## Declaration of interest's statement

The authors declare no conflict of interest.
